# High estradiol and low testosterone levels are associated with critical illness in male but not in female COVID-19 patients: a retrospective cohort study

**DOI:** 10.1080/22221751.2021.1969869

**Published:** 2021-09-14

**Authors:** Maria Schroeder, Berfin Schaumburg, Zacharias Mueller, Ann Parplys, Dominik Jarczak, Kevin Roedl, Axel Nierhaus, Geraldine de Heer, Joern Grensemann, Bettina Schneider, Fabian Stoll, Tian Bai, Henning Jacobsen, Martin Zickler, Stephanie Stanelle-Bertram, Kristin Klaetschke, Thomas Renné, Andreas Meinhardt, Jens Aberle, Jens Hiller, Sven Peine, Lothar Kreienbrock, Karin Klingel, Stefan Kluge, Guelsah Gabriel

**Affiliations:** a Department of Intensive Care Medicine, University Medical Center Hamburg-Eppendorf, Hamburg, Germany; b Department for Viral Zoonoses-One Health, Leibniz Institute for Experimental Virology, Hamburg, Germany; c Department of Biometry, Epidemiology and Information Processing, University of Veterinary Medicine Hannover, Hannover, Germany; d Institute for Clinical and Laboratory Chemistry, University Medical Center Hamburg-Eppendorf, Hamburg, Germany; e Institute of Anatomy and Cell Biology, Justus-Liebig University of Giessen, Giessen, Germany; f Department of Endocrinology, Diabetology, Obesity and Lipids, University Medical Center Hamburg-Eppendorf, Hamburg, Germany; g Institute for Transfusion Medicine, University Medical Center Hamburg-Eppendorf, Hamburg, Germany; h Institute for Pathology and Neuropathology, University Hospital Tuebingen, Tuebingen, Germany; i Institute for Virology, University for Veterinary Medicine Hannover, Hannover, Germany; j German Center for Infection Research (DZIF), Braunschweig, Germany

**Keywords:** SARS-CoV-2, COVID-19, sex differences, sex hormones, cytokines, critical illness

## Abstract

Male sex was repeatedly identified as a risk factor for death and intensive care admission. However, it is yet unclear whether sex hormones are associated with disease severity in COVID-19 patients. In this study, we analysed sex hormone levels (estradiol and testosterone) of male and female COVID-19 patients (*n* = 50) admitted to an intensive care unit (ICU) in comparison to control non-COVID-19 patients at the ICU (*n* = 42), non-COVID-19 patients with the most prevalent comorbidity (coronary heart diseases) present within the COVID-19 cohort (*n* = 39) and healthy individuals (*n* = 50). We detected significantly elevated estradiol levels in critically ill male COVID-19 patients compared to all control cohorts. Testosterone levels were significantly reduced in critically ill male COVID-19 patients compared to control cohorts. No statistically significant differences in sex hormone levels were detected in critically ill female COVID-19 patients, albeit similar trends towards elevated estradiol levels were observed. Linear regression analysis revealed that among a broad range of cytokines and chemokines analysed, IFN-γ levels are positively associated with estradiol levels in male and female COVID-19 patients. Furthermore, male COVID-19 patients with elevated estradiol levels were more likely to receive ECMO treatment. Thus, we herein identified that disturbance of sex hormone metabolism might present a hallmark in critically ill male COVID-19 patients.

## Background

The current SARS-CoV-2 (Severe Acute Respiratory Syndrome Coronavirus Type 2) pandemic continues taking its toll on human health resulting in 4.1 million lives lost worldwide (as of 20 July 2021). The clinical spectrum of SARS-CoV-2 infection is broad, ranging from mild upper respiratory illnesses to severe primary pneumonia with respiratory failure, multi-organ failure and death [1]. Retrospective cohort studies revealed risk factors associated with disease severity and death. A study from Wuhan, China, which enrolled 191 inpatients on hospital admission since its first occurrence in China in December 2019, reported that older age and comorbidities, such as hypertension, diabetes and coronary heart diseases being among the top three, present poor prognostic markers at an early stage [1]. Another study from the UK linked 10,926 COVID-19-related deaths pseudonymously to primary care records of 17 million individuals, identifying being male, older age, diabetes, asthma, obesity as well as chronic heart diseases among the comorbidities associated with COVID-19 related death [2,3].

Thus, there is increasing evidence that being male constitutes a major risk factor associated with SARS-CoV-2 fatality. However, the underlying factors of sex disparity observed in COVID-19 remain unclear yet. We have recently shown using the golden hamster model, that SARS-CoV-2 infection attacks the reproductive organs and causes massive dysregulation of sex hormones in infected male and female animals [4]. Thus, we wanted to study whether the observed dysregulation in sex hormones upon SARS-CoV-2 infection is also present in COVID-19 patients and poses a risk factor for disease severity.

We herein compared sex hormone levels and cytokine responses in critically ill male and female COVID-19 patients to critically ill non-COVID-19 patients as well as to further control cohorts (healthy individuals and patients with coronary heart diseases due to the high prevalence in critically ill male COVID-19 patients). Analysis within the critically ill cohort was stratified by sex, age and Sequential Organ Failure Assessment (SOFA) scores.

## Methods

### Study design and participants

This retrospective cohort study includes the first 50 laboratory-confirmed (qRT-PCR) SARS-CoV-2 patients (39 males and 11 females) who were admitted to the Department of Intensive Care Medicine at the University Medical Center Hamburg-Eppendorf from 9 March to 29 April 2020 (herein referred as ICU_COVID-19_). As a COVID-19 negative control cohort, we included 42 patients (27 males and 15 females) with laboratory-confirmed negative SARS-CoV-2 PCR who were admitted to the Department of Intensive Care Medicine at the University Medical Center Hamburg-Eppendorf as a control group (herein referred as ICU_non-COVID-19_).

For controlled analysis, we recruited additional SARS-CoV-2 PCR-negative cohorts. We recruited patients with coronary heart diseases as one of the most prevalent comorbidities in our COVID-19 cohort (Institute for Pathology and Neuropathology, University Hospital Tübingen; 25 males, 14 females; herein referred as CHD cohort). We also recruited healthy donors (Institute for Transfusion Medicine, University Medical Center Hamburg-Eppendorf; 30 males, 20 females; herein referred as HC cohort).

A detailed description of the individual cohorts as well as respective inclusion and exclusion criteria are summarized in Supplementary Figure 1.

### Setting

The University Medical Center Hamburg-Eppendorf is a tertiary care hospital with 1738 hospital beds. The Department of Intensive Care Medicine includes regularly 12 multidisciplinary ICUs with a total of 140 ICU beds. Since the first wave of SARS-CoV-2 pandemic in March 2020, a minimum of two and a maximum of four intensive care units treated simultaneously up to 40 patients suffering from COVID-19 or suspected COVID-19.

### Data collection

The following demographic and clinical variables were collected retrospectively for the COVID-19 and non-COVID-19 cohort from the electronic patient data management system (PDMS) (ICM, Dräger, Lübeck, Germany): age, sex, body mass index, comorbidities, admission type and diagnosis, Acute Physiology and Chronic Health Score (APACHE II) and Simplified Acute Physiology Score II (SAPS II) on admission, Sequential Organ Failure Assessment Score (SOFA) within the first 24 h, classification of Acute Respiratory Distress Syndrome (ARDS) using the Berlin definition [5] and the need for mechanical ventilation and extracorporeal membrane oxygenation. Additionally, we recorded antiviral treatment, as well as supportive and experimental COVID-19 therapies for the COVID-19 cohort. Furthermore, we followed the course of the patients and recorded discharge or death.

### Hormone quantification

A panel of 13 hormones was measured in plasma samples by an external laboratory accredited for measurements of human samples (Labor Lademannbogen, Hamburg, Germany). The panel included total testosterone, free testosterone, dihydrotestosterone, androstenedione, 17-β-estradiol (E2), estrone, sex hormone-binding globulin, thyroid-stimulating hormone, free triiodothyronine (T3), free thyroxine (T4), luteinizing hormone, follicle-stimulating hormone and cortisol. In detail, cortisol, TSH, T4, LH, FSH, TT, E2 and SHBG were analyzed by electro-chemiluminescence immunoassay (ECLIA). Free TT was analysed by enzyme-linked immunosorbent assay and DHY-TT was measured by liquid chromatography–mass spectrometry (LC-MS/MS). Estrone levels were measured using a radioimmunoassay (RIA).

### Cytokine and chemokine measurement

A panel of 27 cytokines and chemokines (eotaxin, fibroblast growth factor (FGF), granulocyte-colony stimulating factor (G-CSF), interferon-γ (IFN-γ), interferon γ-induced protein (IP-10), interleukin-2 (IL-2), interleukin-4 (IL-4), interleukin-5 (IL-5), interleukin-6 (IL-6), interleukin-7 (IL-7), interleukin-8 (IL-8), interleukin-9 (IL-9), interleukin-10 (IL-10), interleukin-12 (IL-12), interleukin-13 (IL-13), interleukin-15 (IL-15), interleukin-17 (IL-17), interleukin-1β (IL-1β), interleukin 1 receptor antagonist (IL-1RA), monocyte chemoattractant protein-1 (MCP-1), platelet-derived growth factor-BB (PDGF-BB), regulated upon activation, normal T-cell expressed and presumably secreted chemokine (RANTES), tumour necrosis factor-α (TNF-α), and vascular endothelial growth factor (VEGF)) was measured in plasma samples of COVID-19 patients. Cytokine and chemokine levels were measured using a Bio-Plex Pro^TM^ multiplex assay (#M500KCAF0Y, Bio-Rad, Feldkirchen, Germany) according to the manufacturer’s instructions in a Bio-Plex200 System with high-throughput fluidics (HTF; Bio-Rad, Feldkirchen, Germany).

### Statistical analysis

Continuous variables are expressed as median with first to third quartile. Categorical variables are given as absolute and relative numbers. The distribution of data was visually interpreted using histograms. Variables were compared between groups (COVID-19 and non-COVID-19) with the Wilcoxon–Mann–Whitney *U* test, Student-*T* test and the Fisher’s exact test as appropriate. All given *p*-values in the tables are of descriptive nature and not adjusted for multiple testing.

Statistical evaluation for quantitative data was performed with two-way-ANOVA using Holm-Šidák correction for multiple comparisons. For non-normal data unpaired Mann–Whitney or Kruskal–Wallis test ignoring any multiple comparisons were used. Statistical significance was defined as *p *< 0.05 (* *p* < 0.05, ** *p* < 0.01 and *** *p* < 0.001).

All statistical evaluations mentioned above were performed with SAS®, version 9.4 TS level 1M5 (SAS Institute Inc., Cary, NC, United States) and IBM® SPSS® Statistics 27. Graphical representation of the data was performed via GraphPad Prism 8 v. 8.4.2 (GraphPad Software, Inc.).

## Results

### Male COVID-19 patients present high estradiol and low testosterone levels on ICU admission

A total of *n* = 292 SARS-CoV-2 PCR-positive patients were admitted to the Department of Intensive Care Medicine, at the University Medical Center Hamburg-Eppendorf from 9 March to 31 May 2021. Of these, *n *= 157 (54%) were patients secondarily admitted from referring intensive care units from all over northern Germany. In total, *n* = 194 (66%) of all COVID-19 patients were male and *n* = 98 (34%) of all COVID-19 patients were female. Thereof, *n* = 104 (36%) patients died; of which *n* = 71 (68%) were male and *n* = 33 (32%) were female. These data are in line with large epidemiological data analysis identifying being male as a high-risk factor for COVID-19 related death [2].

Critically ill COVID-19 patients were compared to the respective control group of critically ill non-COVID-19 patients with negative SARS-CoV-2 PCR (Table 1). All COVID-19 patients admitted to the ICU presented at least one comorbidity with obesity, arterial hypertension, coronary heart diseases and diabetes mellitus type II being among the most frequent (Table 1). Within the COVID-19 cohort, among all comorbidities present, coronary heart diseases (CHD) were slightly more prevalent in male COVID-19 patients (*p* = 0.6) (Table S1). A total of 39 (78%) patients met the ARDS criteria [5] in the COVID-19 group and 12 (28.6%) in the ICU non-COVID-19 group. There were no statistical relevant differences in the need for mechanical ventilation (invasive and non-invasive) in both groups (*p* = 0.856). In the ICU non-COVID-19 group, 14 (33.3%) patients had a suspected bacterial infection on admission to the ICU. CRP was significantly higher in COVID-19 patients (*p* < 0.001). In total, mortality within the COVID-19 cohort was 30% (*n* = 15) compared to 11.9% (*n* = 5) in the non-COVID-19 control group.

**Table 1.** Baseline demographic and clinical characteristics, admission diagnosis and treatment-related variables in critically ill patients with COVID-19 and without COVID-19 (NON-COVID-19).

	COVID-19 *n* = 50		Non-COVID-19 *n* = 42		*p*^a^
	Male *n* = 39 (78)	Female *n* = 11 (22)	Male *n* = 27 (64.3)	Female *n* = 15 (35.7)	.168
Demographics					
Age	63 (58–73)	67 (59–71)	67 (58–75)	71 (69–82)	.015
Body mass index	27.4 (24.8–31.9)	25 (22.8–30.7)	25.2 (23–26.8)	23.5 (18.5–27.4)	.003
Comorbid conditions on admission					
Obesity (BMI>30 kg/m^2^)	11 (28.2)	3 (27.3)	3 (11.1)	2 (13.3)	.321
Arterial hypertension	20 (51.3)	5 (45.5)	19 (70.4)	9 (60)	.371
Bronchial Asthma	1 (2.6)	0	3 (11.1)	0	.350
COPD	0	1 (9.1)	3 (11.1)	1 (6.7)	.093
Coronary heart disease	8 (20.5)	1 (9.1)	6 (22.2)	4 (26.7)	.752
Diabetes mellitus	13 (33.3)	3 (27.3)	5 (18.5)	4 (26.7)	.645
HbA1C V%	6.4 (5.9–7.1)_n = 30_	6.2 (6–7)_n = 9_	6.8_n = 1_	6.5_n = 1_	
ICU stay					
ARDS					
None	9 (23.1)	2 (18.2)	16 (59.3)	14 (93.3)	
Mild	4 (10.3)	1 (9.1)	4 (14.8)	1 (6.7)	
Moderate	16 (41)	3 (27.3)	7 (25.9)	0	
Severe	10 (25.6)	5 (45.5)	0	0	
Respiratory support					
Mechanical ventilation	27 (69.2)	9 (81.8)	14 (51.6)	8 (53.3)	.856
Non-invasive ventilation	0	0	6 (22.2)	2 (13.3)	
High flow oxygen therapy	9 (23.1)	1 (9.1)	4 (14.8)	0	.189
ECMO	5 (12.8)	2 (18.2)	1 (3.7)	0	.217
COVID-19 Treatment					
Lopinavir/Ritonavir	6 (15.4)	2 (18.2)			
Hydrochloroquin	0	1 (9.1)			
Adrecizumab	6 (15.4)	0			
Cytosorb Filtration	3 (7.7)	0			
Suspected bacterial infection on admission			9 (33.3)	5 (33.7)	
Scores					
APACHE II	27 (24–30)	29 (25–32)	23 (17-25)	24 (21-31)	<.001
SAPS II	40 (32–47)	42 (36–56)	36 (30.5-48)_n=21_	39 (34–58)_n=11_	.505
SOFA	7 (4–9)	7 (5–10)	4 (3–7)	5 (3–7)	.001
Laboratory parameters on admission					
White blood cell count (10^9^/L)	9.2 (4.8–12.2)	6.5 (0.4–12.9)	11.7 (7.9–15.9)	11.5 (8.5–15.2)	.009
Thrombocytes (10^9^/L)	204 (70–293)	143 (49–222)	199 (144–239)	258 (154–301)	.237
Procalcitonin (μg/L)	0.4 (0.2–2.3)	0.5 (0.1–1.6)	0.2 (0.1–0.4)_n = 8_	0.7 (0.1–1.6)_n = 3_	.119
C-reactive protein (mg/dl)	200 (105–278)	174 (60–293)	47 (13–106)	26 (18–82)	<.001
Lactate (mmol/L)	1.1 (0.8–1.6)	0.7 (0.6–1)	1 (0.8–1.5)	1.4 (0.7–1.9)	.36
Outcome					
Recovered	27 (69.2)	8 (72.7)	24 (88.9)	13 (86.7)	
Deceased	12 (30.8)	3 (27.3)	3 (11.1)	2 (13.3)	

Note: Continuous variables are given as median (1st to 3rd quartile), categorical variables are given as *n* (%).

^a^ Wilcoxon–Mann–Whitney *U* or student *T*-test was performed for continuous variables, as appropriate and Fisher’s exact or Chi-Square test for categorical variables. APACHE II: Acute Physiology and Chronic Health Score, COPD: Chronic obstructive pulmonary disease, COVID-19: Corona Virus Disease 2019, ECMO: Extracorporeal membrane oxygenation, SAPS II: Simplified Acute Physiology Score II, SOFA score: Sepsis-related organ failure assessment score

**Figure 1.**
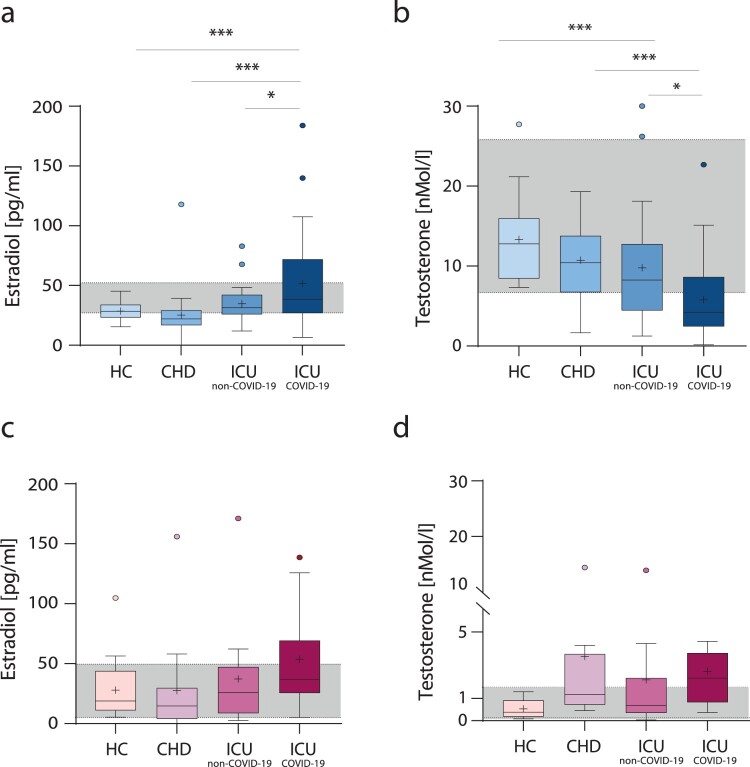
Sex hormone levels in COVID-19 patients, non-COVID-19 patients, patients with coronary heart diseases and healthy individuals. Estradiol (a,c) and testosterone (b,d) levels were measured in plasma obtained from critically ill COVID-19 patients (ICU_COVID-19_), critically ill non-COVID-19 patients (ICU_non-COVID-19_), patients with coronary heart diseases (CHD) and healthy individuals (HC). Male data sets are shown in blue color-toned columns and female data sets are shown in red color-toned columns. The laboratory assessed hormone reference ranges are indicated in grey. Percentile boxplots represent 25–75% of values, with the median value indicated by a crossline, and mean values by a plus icon. Statistical significance was assessed via one-way-ANOVA.

First, we wanted to assess whether sex hormone levels are altered within the critically ill male and female COVID-19 patients potentially providing a link to sex-dependent disease severity. Since CHD was more prevalent in male COVID-19 patients compared to female COVID-19 patients (Table S1), we recruited an age – and sex-matched SARS-CoV-2 negative CHD control cohort (*n* = 39). As an additional healthy control group, we recruited age-and sex-matched SARS-CoV-2 negative male blood donors (HC) (*n* = 50). Furthermore, we recruited SARS-CoV-2 PCR-negative ICU patients (*n* = 42) as an additional control for critical illness independently of COVID-19 (herein referred to as ICU non-COVID-19). Critically ill male COVID-19 patients presented the highest estradiol levels compared to critically ill male non-COVID-19 patients (*p* = 0.0293) or male patients with CHD (*p* = 0.0004) or healthy males (*p* = 0.001) (Figure 1(a)). In line, comparing to published clinical reference values, estradiol levels in critically ill male COVID-19 patients were far above clinical references [6,7]. No significant difference in estradiol levels was detected within the control cohorts, which were within clinical reference values. Conversely, testosterone levels were lowest in critically ill male COVID-19 patients compared to critically ill male non-COVID-19 patients (*p =* 0.0250) or male patients with CHD (*p* = 0.0050) or healthy males (*p* < 0.0001) (Figure 1(b)). In accordance, testosterone levels of most critically ill male COVID-19 patients were below clinical reference values. No significant differences in testosterone levels were detected within the HC and CHD control cohorts. Critically ill female COVID-19 patients presented a trend towards elevated estradiol levels, albeit statistically not significant (Figure 1(c)). Testosterone levels were not significantly altered in critically ill female COVID-19 patients compared to female control cohorts (Figure 1(d)).

Collectively, these findings show that male COVID-19 patients present significantly increased estradiol and reduced testosterone levels compared to non-COVID-19 males. In contrast, female COVID-19 patients do not present significantly altered sex hormone levels.

### Majority of critically ill male COVID-19 patients present secondary hypogonadism

To shed light on the origin of severe testosterone deficiency in male COVID-19 patients, we further analysed related hormones (Table 2). Free testosterone levels were reduced in 66.7% of male COVID-19 patients compared to reference values. Conversely, 54.5% of female COVID-19 patients presented elevated levels of free testosterone. Thus, changes in total testosterone levels correlate with levels of free bioavailable testosterone levels in the respective sex. We then measured levels of the sex hormone-binding globulin (SHBG), since the majority (98%) of total testosterone is bound to SHBG and only 2% is in its free, bioavailable form. Thus, in some cases, testosterone deficiencies might be masked by elevated SHBG levels. In 28.2% of male COVID-19 patients, SHBG levels were elevated, which might suggest masked testosterone deficiencies in some patients. Luteinizing hormone (LH) levels were elevated in 30.8% of male COVID-19 patients, while being within the normal range in all female patients. Interestingly, 7 out of the 28 male patients with low total testosterone levels presented elevated LH levels at the same time (data not shown), suggesting impairment of Leydig cell steroidogenesis in 25% of the male patients. Follicle-stimulating hormone (FSH) levels were elevated in 12.8% of male patients. Elevated FSH levels in these male patients were combined with elevated LH levels. In 45.5% of female patients, FSH levels were reduced, which may indicate loss of ovarian function. This would be in line with the postmenopausal status of the 10 out of 11 COVID-19 females in our cohort. Other hormones, such as thyroid-stimulating hormone (TSH) and T4 were within normal ranges in the majority of male and female patients. Cortisol levels were elevated in 56.4% of male and 81.8% of female COVID-19 patients.

**Table 2.** Hormone levels in critically ill patients with COVID-19.

	COVID-19 *n* = 50	
	Male *n* = 39 (78)	Female *n* = 11 (22)
Free testosterone pg/mL	2.6 (1.7–3.7)	2.3 (0.9–3.6)
Normal males	13 (33.3)	
20–39 yr: 7–22.7 pg/mL		
40–60 yr: 6.3–17.8 pg/mL		
≥61 yr: 2.5–17.8 pg/mL		
Low (all age groups, below reference)	26 (66.7)	
Normal females		5 (45.5)
40–60 yr: ≤2.3 pg/mL		
≥61 yr: ≤2.1 pg/mL		
High (all age groups, above reference)		6 (54.5)
Sex hormone-binding globulin nMol/L	31.3 (19.2–49.9)	33.2 (22.6–58.6)
Normal males: 10–40 nMol/L	27 (69.2)	
Low: <10 nMol/L	1 (2.6)	
High: 41–100 nMol/L	7 (17.9)	
Very High: ≥101 nMol/L	4 (10.3)	
Normal females: 26–110 nMol/L		7 (63.6)
Low: <26 nMol/L		3 (27.3)
High: ≥110 nMol/L		1 (9.1)
Luteinizing hormone mIU/mL	5.5 (2.8–11.8)	6.5 (2.6–16.4)
Normal males: 0–8.6 mIU/mL	27 (69.2)	
High: ≥8.7 mIU/mL	12 (30.8)	
Normal females: <58.5 mIU/mL		11 (100)
Follicle-stimulating hormone	4.2 (2–9.9)	20.1 (6.5–33.7)
Normal males: 1.5–12.4 mIU/mL	32 (82.1)	
Low: <1.5 mIU/mL	2 (5.1)	
High: 12.5–25 mIU/ml	5 (12.8)	
Normal females: 25.8–134.8 mIU/mL		6 (54.5)
Low: 10–25.7 mIU/mL		5 (45.5)
Thyroid-stimulating hormone µU/mL ^ns^	0.8 (0.4–1.5)	0.45 (0.4–1.5)
Normal: 0.27–4.2 µU/mL	30 (76.9)	7 (63.6)
Low: <0.27 µU/mL	7 (18.0)	3 (27.3)
High: >4.2 µU/mL	2 (5.1)	1 (9.1)
Free T4 ^ns^	13.2 (11.2–14.7)	11.1 (10.3–15.1)
Normal: 8–17 ng/dL	38 (97.4)	11 (100)
High: >17 ng/dL	1 (2.6)	
Cortisol µg/dlL^ns^	20.6 (14.8–26.5)	26.7 (14.8–26.5)
Normal: 2.5–19.5 µg/dL	16 (40.0)	2 (18.2)
Low: <23 µg/dL	1 (2.6)	6 (54.5)
High: 23–30 µg/dL	22 (56.4)	9 (81.8)

Note: Continuous variables are given as median (1st to 3rd quartile), categorical variables are given as *n* (%). ^ns^Wilcoxon–Mann–Whitney *U*-test was performed; the difference between sexes is not significant.

These findings suggest that in 25% of the male COVID-19 patients with low total testosterone levels, testosterone deficiency is likely of testicular origin. Thus, in 75% of male patients, the origin of testosterone deficiency is yet unclear.

### Male COVID-19 patients present high estrone and low DHT levels on ICU admission

Testosterone is further metabolized to dihydrotestosterone by 5-α reductase. Dihydrotestosterone (DHT) also acts as an androgen and plays a key role in activating the transcription of various genes and activation of various immune cells similar to testosterone [8]. Thus, we wanted to assess whether alterations in testosterone levels detected in male COVID-19 patients are also reflected in its most potent metabolite. In male COVID-19 patients, dihydrotestosterone levels were reduced compared to HC males (*p* < 0.0001) (Supplementary Figure 2a). In line, a substantial proportion of plasma dihydrotestosterone levels in COVID-19 males was even below the lowest reference range, confirming dihydrotestosterone deficiency in men. In contrast, dihydrotestosterone levels were comparable and within clinical references in female COVID-19 and HC cohorts (*p* = 0.9568) (Supplementary Figure 2c). To assess whether the increase in estradiol levels can be attributed to a general increase in estrogens, we next measured estrone concentrations. Estrone levels in the plasma of COVID-19 males were higher than those measured in HC males (*p* < 0.0001) (Supplementary Figure 2b). Similarly, estrone levels were elevated in the plasma of female COVID-19 patients unlike HC females (*p* = 0.0009) (Supplementary Figure 2d).

Critically ill male COVID-19 patients present additionally elevated estrone levels accompanied by severely reduced DHT levels. Critically ill female COVID-19 patients present elevated estrone levels, while dihydrotestosterone levels remain unchanged.

### Male COVID-19 patients with high estradiol levels are more likely to require ECMO treatment

**Figure 2.**
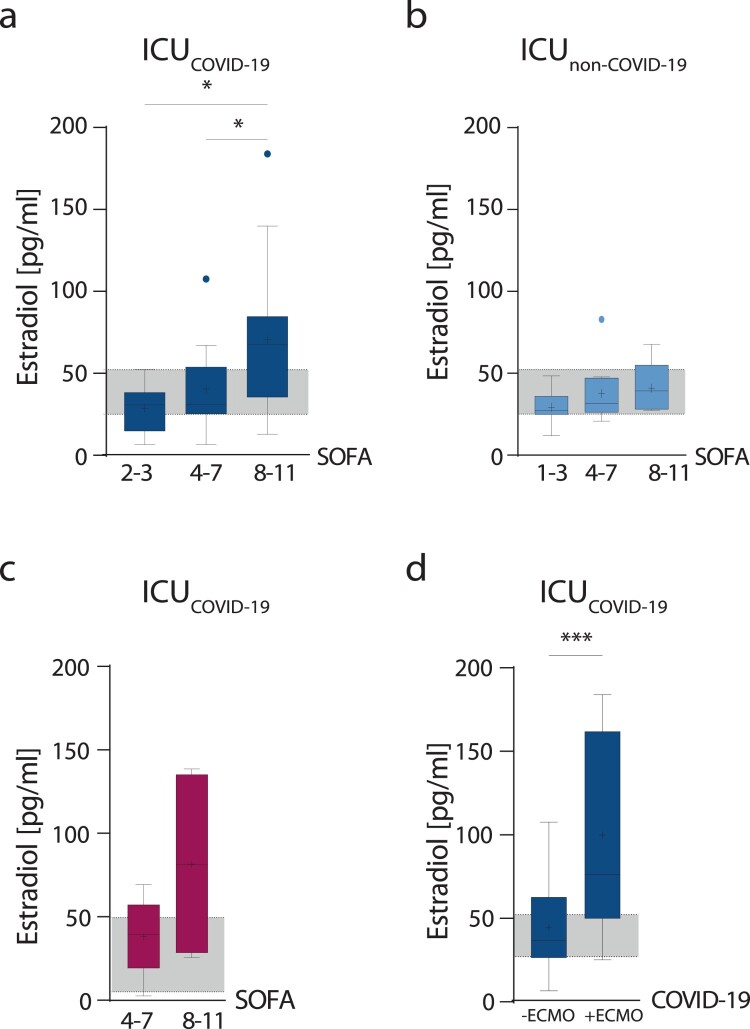
Estradiol levels in dependency of disease severity. Estradiol levels measured in plasma obtained from critically ill male (blue columns) and female (red columns) COVID-19 or non-COVID-19 patients are displayed in dependency of disease severity as assessed by SOFA scores (a–c). Male COVID-19 patients were subdivided into patients requiring ECMO therapy (+ECMO) and patients not requiring ECMO therapy (-ECMO) (d). Percentile boxplots represent 25–75% of values, with the median value indicated by a crossline, and mean values by a plus icon. The laboratory assessed hormone reference ranges are indicated in grey. Values are shown as median and interquartile ranges. Statistical significance in males was assessed by NON-parametric tests (Kruskal–Wallis test and Dunn’s test for multiple comparisons). Statistical significance in females was evaluated by unpaired, two-tailed NON-parametric Student’s *t*-test (Mann–Whitney test).

Next, we analyzed whether sex hormone levels correlate with an increased risk for severe disease outcome as assessed by the Sequential Organ Failure Assessment Score (SOFA) scores or the requirement for extracorporeal membrane oxygenation (ECMO) later during the patients’ ICU stay. In critically ill male COVID-19 patients, estradiol levels increased with disease severity (Figure 2(a)) unlike in non-COVID-19 males (Figure 2(b)) (*p* = 0.0245 and *p* = 0.0273). In female COVID-19 patients, a trend towards higher estradiol levels with increasing SOFA scores was also observed (Figure 2(c)). Male COVID-19 patients with elevated estradiol levels were more likely to require ECMO treatment than those having estradiol levels within normal clinical references (*p* = 0.0307) (Figure 2(d)). Within the female COVID-19 cohort, only one patient required ECMO treatment; thus, not allowing statistical analysis (Table 1). Testosterone levels did not show statistically significant changes in dependence of SOFA scores or patients requiring ECMO treatment (Supplementary Figure 3a-d). This might be due to the fact that most male patients presented low testosterone levels below clinical references [6,7].

These data show that critically ill male COVID-19 patients with elevated estradiol levels are more likely to require ECMO treatment.

### Estradiol levels are positively associated with IFN-γ expression in male COVID-19 patients

**Figure 3.**
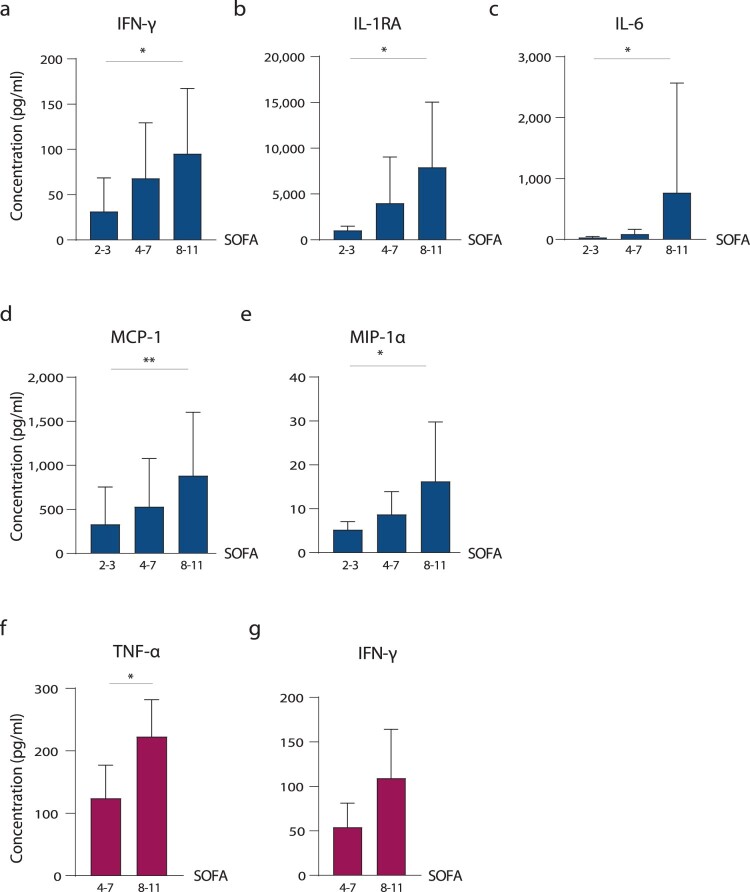
Chemokine and cytokine responses in COVID-19 patients. Cytokine and chemokines were measured in plasma obtained from critically ill male (a–e) and female (f and g) COVID-19 patients using a 27-plex immunoassay. Those with significant differences are shown. Cytokine and chemokine levels of male and female COVID-19 patients are displayed in dependency of disease severity as assessed by SOFA scores (2–3; 4–7; 8–11).

To assess whether altered sex hormone levels might be associated with changes in innate immune responses, we compared cytokine and chemokine patterns in male and female COVID-19 patients. First, we analysed a panel of 27 different cytokines and chemokines in the plasma of COVID-19 patients and correlated these to SOFA scores (Figure 3, Supplementary Figure 3e-m). In male COVID-19 patients, IFN-γ (*p* = 0.0301), IL-1RA (*p* = 0.0160), IL-6 (*p* = 0.0145), MCP-1 (*p* = 0.0052) and MIP-1α (*p* = 0.0134) levels were elevated in those with higher SOFA scores (8-11) compared to those with lower SOFA scores (2-3) (Figure 3(a–e)). In female COVID-19 patients, TNF-α levels were higher in those with high SOFA scores compared to those with low SOFA scores (*p* = 0.0476) (Figure 3(f)). Albeit statistically not significant, IFN-γ showed a trend towards elevation with increasing SOFA scores (Figure 3(g)).

**Figure 4.**
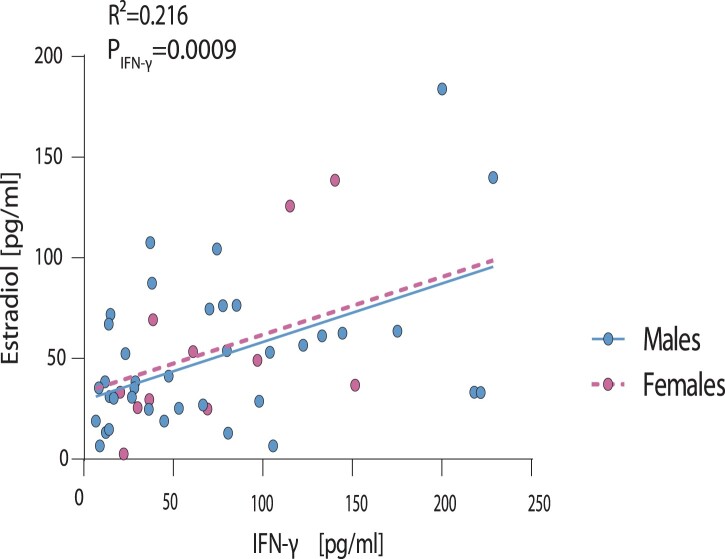
Correlation of IFN-γ levels in male and female COVID-19 patients to estradiol levels. Estradiol levels measured in plasma of critically ill male and female COVID-19 patients were plotted against the expression levels of all cytokines and chemokines assessed. Here, only IFN-γ is displayed, which showed a significant correlation to estradiol levels. Statistical significance was assessed by generalized linear regression.

We then analyse whether changes in cytokine and chemokine responses in critically ill COVID-19 patients correlate with their respective sex hormone levels given that most immune cells possess androgen and estrogen receptors, using multivariable regression [8–10]. Among all 27 cytokine and chemokines assessed, only IFN-γ presented a significant correlation to estradiol in male and female COVID-19 patients (*R*^2^ = 0.216, *p* = 0.0009; Figure 4). Multivariable analysis further revealed that testosterone levels are not associated with changes in IFN-γ levels (*R*^2^ = 0.133, *p* = 0.3111; Supplementary Figure 4).

These findings suggest an association of estradiol levels with IFN-γ levels, which is in line with previous studies reporting on the estradiol-controlled transcription of IFN-γ due to the presence of an estrogen-responsive element (ERE) in its promoter region [11–13].

## Discussion

In this study, we show that disturbance of sex hormone metabolism is associated with critical COVID-19 in men but not in women.

In critically ill men with COVID-19, we detected significantly elevated estradiol levels compared to control cohorts (HC, CHD and critically ill non-COVID-19). Furthermore, testosterone levels were significantly reduced in males with COVID-19 compared to all control cohorts. Our findings were recently confirmed by two independent single-centre studies [14,15]. A study from Italy reported that men with COVID-19 have significantly reduced testosterone and significantly increased estradiol levels compared to healthy controls [15]. Therein, low testosterone levels in COVID-19 males were also associated with secondary hypogonadism and severe outcome in line with our findings reported here [15]. Another study from the US reported that lower testosterone concentrations during hospitalisation were associated with increased disease severity and inflammation in men with COVID-19 [14]. Estradiol levels were not significantly altered in the US study, where severely ill COVID-19 males were directly compared to severely ill non-COVID-19 males. However, it is important to define disease severity very precisely. In our study, we subdivided disease severity using SOFA scores. We found that men with COVID-19 presented a step-wise increase in estradiol levels with increasing SOFA scores unlike men without COVID-19. Furthermore, when comparing all cohorts, we identified estradiol to be a predictive marker for later ECMO requirement during the hospital stay in males with critical COVID-19.

Reduced testosterone levels were likely of testicular origin in 25% (primary hypogonadism) of all male COVID-19 cases in our study, suggesting that 75% can likely be attributed to hypothalamic-hypopituitary origin (secondary hypogonadism). These findings are further confirmed by the Italian single-centre study reporting that low testosterone levels in men with COVID-19 are associated with secondary hypogonadism [15]. The vast majority (95%) of testosterone is produced in Leydig cells of the testes depending on stimulation by luteinizing hormone (LH). Only small amounts (5%) are produced in the adrenal glands. Low levels of testosterone may either be of testicular origin (primary hypogonadism), of hypothalamic-pituitary origin (secondary hypogonadism) or a combination of both, with the latter predominantly found in the aging male population as late-onset hypogonadism [16,17]. Hypogonadism with and without elevated estradiol levels was reported before in patients with cardiovascular diseases as a risk factor for increased mortality in men [18–20]. Thus, it is tempting to speculate whether an initial comorbidity-driven hit, with respect to low testosterone levels, which are also reported in patients with obesity and type II diabetes [21,22] might put males at higher risk to develop severe COVID-19.

In women with COVID-19, we did not detect any significant alterations in testosterone or estradiol levels. However, some female COVID-19 patients had elevated testosterone levels albeit statistically not significant. It is important to note that all but one female COVID-19 patients were postmenopausal. However, the small female COVID-19 cohort size in our study is a potential limitation with respect to conclusions on female COVID-19 outcome. This variance in cohort size was due to that more men than women were admitted to the ICU, highlighting the importance of sex on critical COVID-19 outcome. Despite these limitations in the female COVID-19 cohort, others postulated an elevated COVID-19 risk for women with polycystic ovary syndrome, a condition characterized by increased androgen levels [23]. This highlights the need for further investigations to understand the impact of elevated testosterone levels in women in the context of COVID-19.

Comparative analysis of 27 different cytokines/chemokines in the male and female COVID-19 patients and subsequent regression analysis revealed that interferon-γ (IFN-γ) positively correlates with estradiol but not testosterone levels. This correlation is particularly interesting given that IFN-γ possesses an estrogen-responsive element (ERE) in its promoter region [11–13]. IFN-γ is a key activator of macrophages [13,24] and macrophage activation was repeatedly reported as a hallmark of COVID-19 severity [25]. Macrophages contain membrane-bound as well as nuclear androgen- and estrogen-receptors. Thus, it will be of high interest to dissect the role of sex hormones and IFN-γ in orchestrating, e.g. macrophage activation and cytokine storm in future studies as outlined in a hypothesis model (Supplementary Figure 5).

Collectively, our findings herein highlight that disturbance in circulating sex hormone levels is a hallmark of critical COVID-19 in males. Longitudinal hormone surveillance studies may be warranted during acute and recovery phases in COVID-19 patients to predict short- and potentially long-term risks [26–29].

**List of abbreviations**
APACHE II: Acute Physiology and Chronic Health Score
ARDS: Acute Respiratory Distress Syndrome
CHD: Coronary heart diseases
COVID-19: Coronavirus disease 2019
DZIF: German Center for Infection Research
E2: 17-β-estradiol
ECLIA: Electro-chemiluminescence immunoassay
ECMO: Extracorporeal membrane oxygenation
ERE: Estrogen responsive element
HC: Healthy Control
ICU: Intensive Care Unit
IFN-γ: Interferon-γ
IQR: Interquartile range
LH: Luteinizing hormone
PCOS: Polycystic ovary syndrome
PDMS: Patient data management system
RIA: Radioimmunoassay
SAPS II: Simplified Acute Physiology Score II
SARS-CoV-2: Severe Acute Respiratory Syndrome CoronavirusType 2
SHBG: Sex hormone-binding globulin
SOFA: Sequential Organ Failure Assessment Score
T3: Free triiodothyronine
T4: Free thyroxine
UKE: University Medical Center Hamburg-Eppendorf
WHO: World Health Organization

## Ethical approval and consent to participate

The study with laboratory-confirmed COVID-19 and non-COVID-19 ICU patients was reviewed and approved by the ethics committee at the Hamburg State Chamber of Physicians (registration no.: WF-053/20 and WF-073/21). The need for an informed consent for the ICU cohort, healthy blood donors (HC) and patients with coronary heart diseases (CHD) was waived by the ethics committee because data were retrieved anonymously and retrospectively from electronic health records. The study is registered under the number NCT04979091 (HAM-SEX-C19) on ClinicalTrials.gov.

## Supplementary Material

Figure_S5_EMI.eps

Figure_S4_EMI.eps

Figure_S3_EMI.eps

Figure_S2_EMI.eps

Figure_S1_EMI_revised.eps
